# Postbiotics of Naturally Fermented Synbiotic Mixture of Rice Water Aids in Promoting Colonocyte Health

**DOI:** 10.3390/biom14030344

**Published:** 2024-03-13

**Authors:** Charumathi Anbalagan, Sangeetha Kadapakkam Nandabalan, Pavithra Sankar, Prasanna Srinivasan Rajaram, Karthick Govindaraj, Secunda Rupert, Jeswanth Sathyanesan

**Affiliations:** Department of Regenerative Medicine and Research, Government Stanley Medical College and Hospital, Chennai 600001, India; acharuonline@gmail.com (C.A.);

**Keywords:** fermented rice water (FRW), synbiotic mixture, probiotics, Lactic Acid Bacteria (LAB), postbiotics, metabolomics, microbial identification, colonocyte health, metabolites

## Abstract

The eubiotic state of the gut microbiota is primarily brought about by various probiotic species that colonize the gut. It is becoming very clear that the probiotic-metabolite mixtures in the gut luminal milieu is central in establishing cross-kingdom signalling networks to maintain gut-multi-organ axes health. Culturally, different fermented foods and beverages have been regional staples since ancient times, and are known to be enriched with probiotics. However, regional variations including the environment, the staple food source (prebiotics), and fermentation methods, among other factors, influence the fermenting probiotic species. Fermented rice water (FRW), an economical, easy to make, simple beverage is a rich source of synbiotics. Therefore, consumption of fermented rice water allows for the intake of a variety of region-specific live probiotics. The secondary metabolites (postbiotics) present in such symbiotic mixtures may also contribute toward maintaining normal intestinal cellular functions. In this study, we highlight that regional staples such as rice consumed in their fermented form may hold promise in alleviating gut-related diseases. Our results show that simple overnight fermentation of cooked edible rice enables the growth of probiotic bacterial species belonging to the Lactic Acid Bacteria group (*Leuconostoc lactis*, *Weisella confusa*, *Weisella cibacria, Lactococcus lactis*, *lactococcus taiwanensis*, *Lactobacillus fermentum*, *Lactobacillus nagelii*, and *Lactobacillus delbrueckii* ssp. *indicus*). Metabolomic analysis of the overnight fermented and over two-nights fermented rice water identified more than 200 postbiotic metabolites. Our results show that postbiotics contributing to energy metabolism, gut-multiorgan axes, and microbial paraprobiotics are enriched in the overnight (~10 h) fermented rice water as compared to the over two-nights fermented rice water. Functional analysis via gene expression studies for nutrient absorption (mct-1 and mct-2) and barrier integrity (occludin and zo-1) reveals significant upregulation of these genes upon FRW treatment of HT29 colon cells. This study is a first-of-its-kind to demonstrate the proof-of-principle that postbiotics of naturally fermented rice water positively modulates colonocyte health.

## 1. Introduction

It has become evident from research in several species of the animal kingdom that the gut microbiota (bacterial ecosystem) is central in maintaining a healthy state [1,2,3,4,5]. The composition of the microbiota is different in animal species due to their inherent genetic constitution, and is primarily linked with the food chain. Importantly, the microbiota of humans also greatly varies between different races, and even within a given population. It is also known that cross-kingdom microbiota links brought about by anthropogenic and environmental factors play a major role in establishing this unique microbiome in humans. Irrespective of variations in the microbiota that colonize our gut, there is an underlying microbiological-ecosystem composed of a sect of microbial species that establishes a healthy gut-organismal status. These are the probiotic species which are established right from birth, continuing to modulate the gut microbiome throughout the adult phase [6]. Perturbations in the composition of the normal microbiota leads to dysbiosis of the gut, which is characterized by an imbalance in the beneficial versus the pathogenic microbial populations [7]. Gut dysbiosis has both local and systemic implications in health and disease.

Although pharmaceutical interventions help alleviate the symptoms of gut dysbiosis, it is only intuitive to think that setting the microbial ecosystem right must be the first choice of intervention. Naturally, there is a strong connection between the dietary intake and the microbiome in the gut, where food components greatly influence the microbiota [8]. Fermented foods are a rich source of probiotic bacteria. Although probiotic supplements are commercially available that contain a mixture of different strains of lyophilized bacteria, consumption of fermented food allows for intake of live probiotics (billions of colony-forming units) along with the symbiotic mixture. Several studies have highlighted the role of probiotics in restoring intestinal homeostasis [1,9,10]. Probiotic bioactives have been implicated in modulating several cellular signalling pathways in response to the environmental stimuli [11,12,13,14,15]. It is known that there is a rich diversity and distribution of microbial species throughout the intestinal system. However, the benefits of probiotics are primarily felt in the anaerobic regions of the colon. Hence, chronic consumption of probiotics alone may be able to restore the eubiotic state. However, consumption of synbiotics and postbiotics may boost their benefits [16].

Intestinal health, and by extension organismal health, is directly linked to the consumption of food. Of late, the role of gut microbiome, especially a rich and a diverse microbiome, in influencing a broad spectrum of human diseases is intensively studied [17,18,19]. The main contributor of diversity of the microbiome in the gut is the consumption of fibre-rich foods [20]. This allows for enrichment of the probiotic species present in the gut. Also, consumption of fermented foods has been practised in almost all cultures to improve gut health. Recently, a plethora of scientific evidence in support of the role of fermented foods in maintaining gut health has led to the translation of folklore beliefs in their use as nutraceuticals and biotherapeutics [21,22]. One such practice is the consumption of fermented rice along with its water in south Asian countries.

Synergistic effects of pre-, pro-, and post-biotics are paramount in maintaining an eubiotic state [23,24,25]. However, postbiotics have emerged as a class of molecules that bring about a plethora of health benefits through several modes [26]. Postbiotics are either inanimate microorganisms or their cellular components and their metabolites, which are beneficial to the host [27]. The role of postbiotics in managing and mitigating several systemic diseases including metabolic, mental health, cancer and gut-liver disorders have been explored [28,29,30,31,32,33]. Nevertheless, direct influence of the postbiotics is first seen in the gut, where the gut-resident bacteria utilize postbiotics either as their energy source(s) or for further alteration of the microbiome [34,35,36,37]. For example, it is shown that administration of heat-killed lactobacilli as postbiotic, render bifidogenic effects in the gut [38]. Several studies indeed have shown that fermentation of a wide variety of foods, such as whey, citrus pomace extract, and oats, by the lactic acid bacterial group generate postbiotics (polyphenols) that cause trans-acting effects in the host’s organs [34,39,40]. Recently, it has been shown that the gut microbiome establishes a gut-skeletal muscle axis via the postbiotic metabolites (acetate and succinate) by increasing muscle energetics through mitochondrial respiration during exercise [41]. Combinative therapies with a commercially available tyndallized mixture of postbiotics (*Bifidobacterium longum* and *Lactobacillus acidophilus* lysates) along with exercise has been shown to improve even brain function in Alzheimer’s disease mice models [32]. Overall, it is very clear that the postbiotic consortia plays an important role in maintaining gut-multi organ health.

In this study, we investigated the role of fermented rice water containing the postbiotic biomolecules in modulating the colonocyte health in vitro, using the HT29 cell line as a model. To this end, we fermented 3 different rice varieties of regional staples at four different locations, and identified the presence of probiotics in all the fermented rice products. We further show that the resultant bioactives positively modulate the colonocytes.

## 2. Materials and Methods

### 2.1. Chemicals, Reagents, and Growth Media

Cell culture media and supplements such as Dulbecco’s Modified Eagle Medium (DMEM) containing low glucose (Cat. No. D6046) /high glucose (Cat. No. 5671) and l-glutamine (Cat. No. 59202c) were procured from Sigma-Aldrich (Darmstad, Germany). Trypsin-EDTA (Cat. No. 25200072) (Gibco (Bangalore, India), source-porcine pancreas) was purchased from Thermo Fisher Scientific. Fetal Bovine Serum (FBS) (Cat. No. 16000044) of USA origin was purchased from Gibco. Trizol (Cat. No. T9424) for RNA extraction, DMSO (Cat. No. D5879) for cell culture, Sodium butyrate (Cat.No. 303410) was purchased from Sigma and real time primers were procured from Sigma (Bangalore, India). MTT reagent (Cat. No. RM1131) was procured from Himedia (Mumbai, India). cDNA synthesis kit (Cat. No. 1708891) from BIORAD (Hercules, CA, USA). Microbial cell culture media such as Nutrient agar (NA) (Cat. No. M001) and De Man, Rogosa, and Sharpe agar (MRS) (Cat. No. M641) were purchased from Himedia.

### 2.2. Preparation of Fermented Rice Water (FRW) and Non-Fermented Rice Water (nFRW) from the South Indian Rice Varieties

Three different south Indian rice varieties (White Ponni (WP), Raw Rice (RR), and Mappillai Samba (MS)) (Figure S2) were used in this study. Respective rice varieties (100 g) were washed thoroughly, soaked and cooked on a stove-top. The cooked rice was cooled completely and to the cooled rice 300–400 mL of potable water (reverse osmosis purified) was added. The rice and the water mixtures were allowed to ferment in earthen pots overnight. Likewise, for the non-fermented rice water sample, a matched setup was followed as in the case of fermented rice water with the addition of antibiotics penicillin/streptomycin at 10,000 units (cat no. 10378016) and sodium azide 1 mM (cat no. S2002) during the overnight fermentation step to prevent fermentation. Also, to ensure that fermentation is facilitated, it is mandatory to cover the earthen pot with tight lid (to maintain anerobic condition).

### 2.3. Proximate Composition Analysis

Macronutrient changes in the different rice varieties (White Ponni, Ration Rice and Mappillai Samba) were determined quantitatively by proximate analysis following the Association of Official Analytical Chemists (AOAC, 2000) methods for the parameters (carbohydrate, fats, protein and ash). The crude protein content of the rice samples was determined with the Kjeldhal method for determining the total nitrogen content and the obtained nitrogen values were multiplied by 6.25 to convert it to crude protein. The ash and the moisture content were determined by gravimetric method. The crude fat of the rice samples was determined based on acid hydrolysis method with petroleum ether as solvent using Soxhlet extractor at 60 °C following the AOAC procedure. Finally, the total carbohydrate content was determined based on the difference method calculation (AOAC, 2000).

### 2.4. Microbial Culture for Isolation, Enumeration and Identification of the Microbial Species Grown in FRW

To identify the fermenting bacterial species in the respective overnight and over two-nights fermented rice water from the different rice varieties, an aliquot of the FRW sample from the respective rice varieties were collected individually in sterile containers and processed by following the aseptic microbiological plating techniques. The samples were plated in both De Man, Rogosa, and Sharpe agar (MRS) (cat no. M641) and Nutrient agar (NA) (cat no. M001) and maintained in anerobic and aerobic conditions at 37 °C for 24 h, respectively. The microbial organisms grown in the MRS and NA were sub-cultured as individual colonies and identified using MALDI-TOF instrument. Briefly, a single colony of the organism grown was smeared as a thin film directly onto the target spot on the MALDI plate and allowed for complete drying. After drying, 1 μL of matrix solution (*α*-cyano-4-hydroxycinnamic acid, HCCA (10 mg/mL)) was applied to the target plate and dried at room temperature. The sample plate was read in the MALDI Biotyper sirius one RUO System and identification result was interpreted according to the manufacturer’s recommendations as “reliable species identification” for score 2.00–3.00, “reliable genus identification” for score 1.70–1.99, and “no reliable identification” for score < 1.7. Likewise, as control for the fermentation experiment, i.e., to confirm that the presence of the bacterial species is indeed due to the fermentation process, a matched non-fermenting condition was set up by the addition of Penicillin–Streptomycin cocktail or Sodium Azide, for each rice variety. For the enumeration of probiotics in the FRW samples, serial dilution (10^−1^ to 10^−7^) was performed and the dilutions were spread on the MRS agar plates based on the plate method. The plates were anaerobically incubated at 37 °C for 24 h. Colony forming unit per millilitre (CFU/mL) of Probiotics for was calculated using the formula CFU/mL = (Number of colonies × Dilution factor)/Volume of culture plated.

### 2.5. Lyophilisation of Fermented Samples

Following overnight fermentation, the water from both the rice mixtures (FRW and nFRW) was separated by aspiration or decantation into a sterile 50 mL centrifugal tubes Falcon (cat. no. 352070) and centrifuged at 10,000× *g* for 10 min to remove solids (larger debris and smaller colloidal substances). Finally, the rice water devoid of any solid material, i.e., clear supernatant (20 mL) was transferred to a sterile 50 mL centrifuge tube and placed immediately in a −80 °C deep freezer to freeze the contents. The frozen contents were lyophilized in a (FreeZone^6^) until the moisture was completely removed.

### 2.6. Sample Preparation for Metabolomic Analysis

Lyophilised fermented rice water (FRW) and non-fermented rice water (nFRW) samples of white ponni rice variety were reconstituted to a concentration of 1 mg/mL in LCMS grade water. The reconstituted samples were vortex and sonicated for 2 min and centrifuged at 14,000 rpm for 10 min. Following centrifugation, the supernatant was collected and further diluted with water and methanol (1:1) in the ratio 1:10. 10 mL of the diluted sample was injected into the LCMS equipment.

### 2.7. Data Acquisition

#### 2.7.1. UHPLC

LC-HRMS analysis was performed on an Dionex Ultimate3000 UHPLC system (Dionex, Lohmar, Germany), coupled to a Q-Exactive mass spectrometer (Thermo Fisher Scientific, Bremen, Germany). The chromatography system was equipped separately with two columns: (i) RP-LC (Reverse Phase Liquid Chromatography) Zorbax Sb Aq (5 µm, 3.0 × 150 mm) kept at a constant temperature of 50 °C. The solvent system comprised mobile phase A (ultrapure water) and mobile phase B (Acetonitrile), both acidified with 0.1% formic acid. A constant flow rate of 0.5 mL/min over a run of 30 min. The multistep gradient (followed by 2 min equilibration time) was programmed as follows: 0–1.2 min: 5%B, 2–14 min: 5–55%B, 14–19 min: 55–95%B, 19–23 min: 95%B, 23–23.1 min: 95–5%B, 23.1–30 min: 5%B. (ii) HILIC (Hydrophilic Interaction Liquid Chromatography) Luna (5 µm, 150 × 4.60 mm) kept at a constant temperature of 50 °C. The solvent system comprised mobile phase A (ultrapure water + 10 mM of ammonium acetate) and mobile phase B (acetonitrile) acidified with 0.1% formic acid. A constant flow rate of 0.5 mL/min over a run of 30 min was used with a multistep gradient. The multistep gradient (followed by 2 min equilibration time) was programmed as follows: 0–1.2 min: 5%B, 2–14 min: 5–55%B, 14–19 min: 55–95%B, 19–23 min: 95%B, 23–23.1 min: 95–5%B, 23.1–30 min: 5%B. The auto sampler temperature was set at 4 °C.

#### 2.7.2. MS

ESI (Electrospray Ionization) source was used for both chromatography systems with a spray voltage of 4 kV for positive ion mode and 2.5 kV for negative ion mode and vaporizer temperature of 280 °C, a sheath gas flow of 30 arbitrary units (AU), an auxiliary gas flow of 10 AU operated in positive (ESI+) and negative (ESI−) electrospray ionization modes (one run for each mode).Detection was performed with a full-scan acquisition at 70,000 resolution (*m*/*z* = 200), which ranged from 50.0 to 200.0 *m*/*z.* Xcalibur 2.2 software (Thermo Fisher Scientific, Bremen, Germany) controlled the system.

### 2.8. Reconstitution of the Fermented Rice Water

For the functional analysis, lyophilized FRW and nFRW were initial reconstituted at 10× concentration in DMEM low glucose, 10% FBS supplemented media. The reconstituted samples were the centrifuged at 10,000× *g* for 10 min and the resulting supernatant was then sterile filtered using a 0.22 µm filter to remove the contaminating bacteria. The filtered samples were then used for preparing the other respective dilutions of the samples, i.e., 7.5×, 5×, 2.5×, 1× concentrates. The above-mentioned concentrations of FRW and nFRW were used for dose response and other functional assays mentioned in this study.

### 2.9. Cell Culture

The human colorectal adenocarcinoma cell line (HT29) was procured from National Centre for Cell Sciences (NCCS), Pune, India, at the passage number 6. The cells were cultured in DMEM high glucose medium supplemented with 10% FBS and were maintained at 37 °C in a humidified incubator at 5% CO_2_ with growth media replaced every 2 days. For the experiments, HT 29 cells were maintained in low glucose DMEM supplemented with 10% FBS.

### 2.10. Cell Viability Assay

The viability of HT 29 cells after FRW, nFRW, and sodium butyrate treatment were assessed by MTT assay. HT 29 cells were seeded at a density of 1 × 10^4^ cells per well in a 96 well plate in low glucose containing DMEM supplemented with 10% FBS and allowed to rest until the day of treatment. Then, different concentrations of FRW (10×, 7.5×, 5×, 2.5× and 1×), nFRW (10×, 7.5×, 5×, 2.5× and 1×), and sodium butyrate (−1, −2.5, −5 and −10 µM) were treated and incubated for 24 h and 48 h. Following the respective incubation periods, MTT (5 mg/mL) was added to the cells and incubated for another 4 h. Then, the supernatant was discarded and the formazan crystals formed were dissolved in DMSO (100 µL/well) and allowed for complete dissolution for 10 min at room temperature and read at 630 nm.

### 2.11. mRNA Isolation and cDNA Conversion

For gene expression studies, different concentration of FRW, nFRW, and sodium butyrate (NaB) treated HT29 cells were used for RNA isolation. RNA isolation and cDNA conversion were performed as described in [42].

Briefly, HT29 cells after the above specified treatment condition were homogenised with trizol reagent (1 mL) followed by the addition of 0.2 mL of chloroform (cat no. C2432) was added. The homogenates were allowed to separate into an upper aqueous layer (containing RNA), an interphase and lower organic layer by centrifugation at 12,000× *g* for 10 min at 8 °C. The aqueous layer containing the RNA was aspirated and collected separately and the RNA was precipitated with isopropyl alcohol (IPA) (cat no. I9030) and centrifuged at 12,000× *g* for 10 min. The RNA pellet after centrifugation was washed with 70% ethanol, (cat no. F204325) dried and then re-suspended in RNAse free water and quantified by spectrophotometry at 260/280 nm. The quantified RNA (purity of RNA to be greater than 2.0 at 260/280 nm) was converted to cDNA using cDNA synthesis kit (BIORAD).

### 2.12. Gene Expression Studies Using qPCR

In order to quantitate the gene(s) expression changes between the various treatments, we adopted quantitative real-time polymerase chain reaction (qRT-PCR) using the Applied Biosystems 7500 fast system (Foster City, CA, USA). The reaction was setup with 100 ng of the respective cDNA samples using Quanti Nova SYBR GREEN (Hilden, Germany) (Cat. No. 208054) chemistry. (Primers for Occludin, Zo-1) mct-1 and -2, β-actin and gapdh were obtained from previously published literature. The conditions for qRT-PCR were as follows denaturation at 95 °C for 30 s, annealing (temperature variable for each primer) for 1 min and final extension at 72 °C for 10 min with 40 cycles. T-bet, a T cell specific transcription factor, which did not show any alterations in expression between the treatments was used as an internal control. Data was represented as relative gene expression normalized to internal control gene expression.

### 2.13. Statistical Analysis

The cell proliferation and gene expression data presented in this study are an average of two or more independent experiments. The statistical software package GraphPad Prism 10.2 was used to perform statistical analysis employing ANOVA with Turkey’s or Dunnett multiple comparison. A *p* value of less than <0.05 is considered as statistically significant.

## 3. Results

### 3.1. Natural Fermentation of Edible Rice Enrich for Probiotics

#### 3.1.1. Rice-Based Carbon Source Alone Is Enough for Short-Duration Natural Fermentation

To show that fermentation of edible rice, in general, can considerably enrich gut-beneficial probiotic microbes, we fermented three different, off-the-shelf, commercially available, locally sourced, rice varieties. They are two commonly available generic rice varieties (par-boiled White Ponni (WP), Raw Rice (RR)) and one not so common, traditional variety known as Mappillai Samba (MS). Since it is customary to consume over-night-fermented rice along with its water as breakfast the next day, we also fermented the rice overnight, i.e., limiting the duration of fermentation to approximately 10 to 12 h for most experiments, unless otherwise stated. In order to enable the growth of fermenting bacteria, we followed the simple fermentation procedure mentioned in Section 2.2. Soaking cooked rice in plain water, in either earthen or in non-leaching metal containers under partial anaerobic conditions (with lids on), allows for fermentation. Visible cues of the fermentation process included formation of tiny air pockets at the surface of the rice-water mixture in the fermented container as compared to the non-fermenting control samples (Figure S1).

#### 3.1.2. Natural Fermentation of Edible Rice Enables Growth of Probiotics of the LAB Group

**Figure 1:**
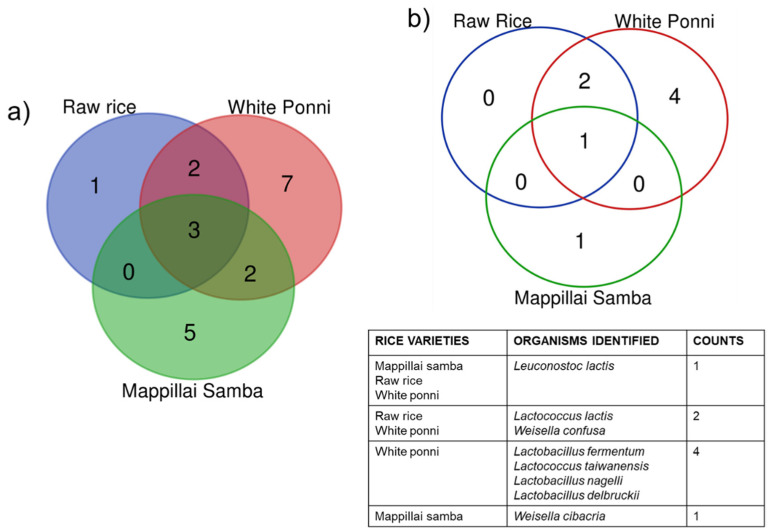
Schematic Venn representations of (**a**) total number of microbial species identified (**b**) probiotic species identified in the three different fermented rice waters at four different environmental locations.

To identify the fermenting bacterial species in the respective fermented rice water (FRW), an aliquot of the FRW was collected in a sterile container and processed by following aseptic microbiological plating techniques as mentioned in Section 2.4. A total of twenty different microbial species were identified between the three different rice varieties. Of which, two different species were present in common between the RR-WP and between WP-MS rice varieties respectively, as shown in the overlaps of the Venn diagram (Figure 1a). Three species were found to be present in all the three rice varieties (Figure 1a). Also, all three rice varieties show rice-specific bacterial growth: RR = 1; WP = 7; MS = 5 (Figure 1a). In order to confirm that the presence of bacterial species in FRW is indeed due to the fermentation process, a matched non-fermenting condition was set up by the addition of Penicillin–Streptomycin cocktail, and/or Sodium Azide, for each rice variety.

Our results show that simple overnight fermentation of cooked edible rice (three different rice varieties) enable the growth of a total of eight different probiotic bacteria. namely *Leuconostoc lactis*, *Weisella confusa*, *Weisella cibacria*, *Lactococcus lactis*, *lactococcus taiwanensis*, *Lactobacillus fermentum*, *Lactobacillus nagelii*, and *Lactobacillus delbrueckii* ssp. *indicus* all belonging to Lactic Acid Bacteria (LAB) group (Figure 1 and Table 1). These eight types of naturally fermenting probiotic bacteria were present among all the three different rice varieties. We also observed differences in the presence of probiotic species identified from the three different FRWs (Figure 1b).

#### 3.1.3. Environmental Influence toward Natural Fermentation of Edible Rice

The respective FRW samples were cultured for microbial growth in both MRS (anaerobic) and Nutrient Agar (aerobic) conditions. Culturing for microbial growth on MRS and NA plates clearly showed selective medium-based differences: LABs in MRS, and commensals or opportunistic pathogens in the NA plates. Understandably, only the hospital environment-fermented rice samples showed the presence of these opportunistic pathogens (Table 2). Interestingly, we identified two species of the *streptococcus* genus belonging to the SBSEC group from the WP-FRW samples fermented at both domicile and Hospital environments (Locations 1 and 4).

### 3.2. Macronutrient Analysis of Each Fermented Rice Variety and Its Water

In order to understand the effect of fermentation on rice and their respective rice waters, when altering the major food group components, we studied the proximate parameters (carbohydrate, fats, protein, and ash) content for the aforementioned three rice varieties.

Our study shows observable changes in crude protein content between the rice forms (before-fermentation, non-fermentation, and after-fermentation) and their respective rice waters. Understandably, solid rice forms contain more crude protein as compared to their respective waters. However, we notice a slight decrease in the crude protein of the after-fermentation rice waters of the Raw rice (5.98%) and (4.97%) Parboiled rice varieties as compared to their before-fermentation (RR, 6.51%; PR, 6.01%) and non-fermentation (RR, 6.88%; PR, 7.30%) samples (Table 3).

We observed an increase in the soluble carbohydrate content (Nitrogen-free extract) of the fermented rice waters of all the three rice varieties (Table 3). The ash content (minerals) was also observed to be increased in the fermented rice water samples. However, the converse is true for the fat content of the fermented rice water samples. Overall, we observe that fermentation of all the three rice varieties lead to a decrease in the crude protein content and increase in soluble carbohydrates and ash content in their respective fermented rice waters (Table 3).

### 3.3. Metabolomic Analysis of the WP-Fermented Rice Water

In order to identify the bioactives present as a result of fermentation, we employed LC-MS/MS omics platform, as described in Section 2. To distinguish the bioactives (postbiotics) of short and long-term fermentation, both overnight- and over-two-nights-fermented rice waters were analysed for their metabolomic composition.

**Figure 2:**
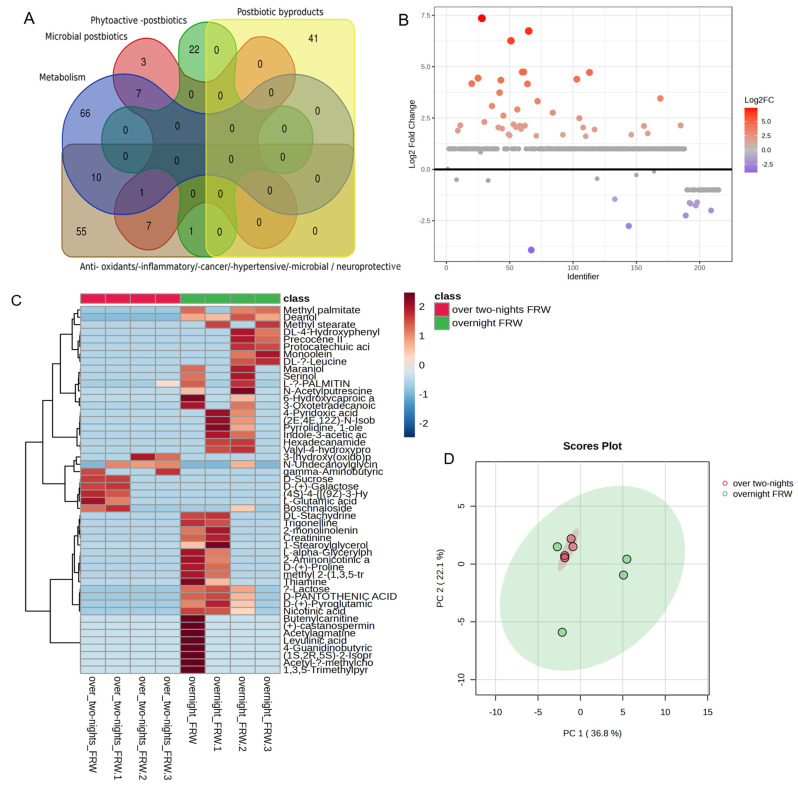

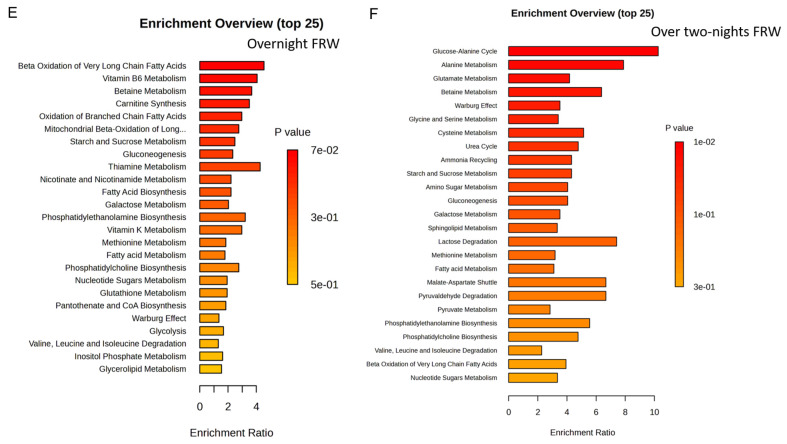
Statistical analysis of metabolomics-derived datasets performed by MetaboAnalyst 6.0. (**A**) Summary of different categories of functionally active metabolites present in fermented rice water (FRW) of the traditional White Ponni rice using Venn diagram generated using the Van de Peer Lab Venn diagram tool (http://bioinformatics.psb.ugent.be/webtools/Venn/) (accessed on 26 February 2024). (**B**) Fold Change (FC) analysis (Unpaired) of overnight /over two-nights FRW. Important features were selected by fold-change analysis with threshold 2. The red circles represent features above the threshold. (**C**) Heatmap showing the unsupervised hierarchical clustering of top 50 metabolites present in overnight-fermented and over-two-nights-fermented rice water (FRW) of White Ponni (WP) rice variety. The colour scale (blue to red) represents the relative abundance of each metabolite. (**D**) Principal Component Analysis (PCA) plot of the total metabolites present in overnightfermented (green dots) and over-two-nights-fermented rice water (FRW) of White Ponni (WP) rice variety (red dots). Green and red ellipses show clear clustering between the groups. (**E**,**F**) Summary Plots for Over Representation Analysis (ORA) of the top 25 enrichment overview of the normal human metabolic pathways (HMDB) for the metabolites of overnight- and over-two-nights-fermented rice water. Dark red bars signify statistically significant over represented metabolic pathways.

We identified over 200 metabolites present in the White-Ponni-fermented rice water (Figure 2A). Fold change analysis of the overnight and over-two-nights fermented rice water reveal 41 significantly upregulated and 9 significantly downregulated metabolites in the overnight fermented rice water as compared to the over two-nights fermented sample (Figure 2B). The metabolites Choline, Lactic acid, and Betaine (top three upregulated) were over 6-fold upregulated while the metabolites Maltose, Boshnaloside, and Glutamic acid (top three downregulated) were 2-fold downregulated in the overnight FRW (Supplementary Figure S5. Metabolites that participate in energy metabolism such as lipid moieties, i.e., long, medium, and short-chain fatty acids were all predominantly present in the overnight fermented rice water sample as compared to the two-nights FRW sample, which is represented in the heat map of the top 50 metabolites (based on peak intensity) (Figure 2C,D). The two-nights FRW predominantly contains simple sugars such as sucrose and galactose (Figure 2C). The scores plot of principal component analysis shows clear clustering of the metabolites present in the two samples. The overlapping eclipses also indicate that there are some similarities in the metabolites present in the FRW samples, i.e., overnight- and over-two-nights-fermented rice water (Figure 2D). The metabolites Lactic acid, Betaine, Azelic acid and Undecanoyl glycine were present in both FRW samples. Interestingly, the two nights FRW contains a high concentration of Guanine GABA.

Postbiotics that render various biological effects, including anti-inflammatory and anti-oxidant properties, were also enriched in the overnight fermented rice water sample as compared to the two nights FRW sample (Figure 2A). Notably, several psycobiotics and neurotransmitters, such as Choline and its derivatives, Gamma Amino Butyric Acid (GABA), Phenyl alanine, and acytlcarnitine, were present in both overnight and over two-nights FRW samples. The ceramide Spinganine is also present in both FRW samples.

Metabolite set enrichment analysis (MESA) of the metabolites of overnight fermented rice water for Over Representation Analysis (ORA) shows enrichment of metabolites that are involved in lipid metabolism, Vit B6 and Betaine metabolism, and mitochondrial beta oxidation of long-chain fatty acids pathways to be (*p* = 0.07) enriched than glycolytic, glycerolipid metabolism and amino acid degradation pathways (*p* = 0.5) (Figure 2E). In contrast, metabolites present in over two-nights fermented rice water are highly enriched for Glucose-Alanine Cycle followed by amino acids metabolism that is statistically significant (*p* ≤ 0.01) (Figure 2F).

### 3.4. Functional Analysis of FRW in the Colonocyte Cell Line HT29

In order to understand how FRW influences the intestinal cellular physiology, we used the human colon adenocarcinoma cell line HT29. Although tumour-derived, these cells are increasingly used in understanding nutrient and bioactives-mediated studies. This is due to their ability to differentiate into nutrient absorptive phenotype under metabolic stress (low glucose) or in the presence of alternate sugars (galactose) and chemical inducers (methotrexate and butyrate).

#### 3.4.1. FRW from Different Rice Varieties Differentially Modulate Proliferation of HT29 Cells

Cell proliferation assay was performed in 96-well plates, as described in Section 2.6. Briefly, HT29 cells were seeded at 10,000 cells per well and were allowed to acclimatize in low glucose DMEM culture medium to maintain an absorptive phenotype. The lyophilized FRW samples were reconstituted and processed as described in Section 2.7. The cells were treated with 2.5-fold increasing concentrations of the reconstituted and 0.22 µm filtered FRW (1× to 10×). On the day of the assay, the transformation of tetrazolium dye (MTT) to the purple-coloured formazan product was measured as a readout as described in Section 2.8.

**Figure 3:**
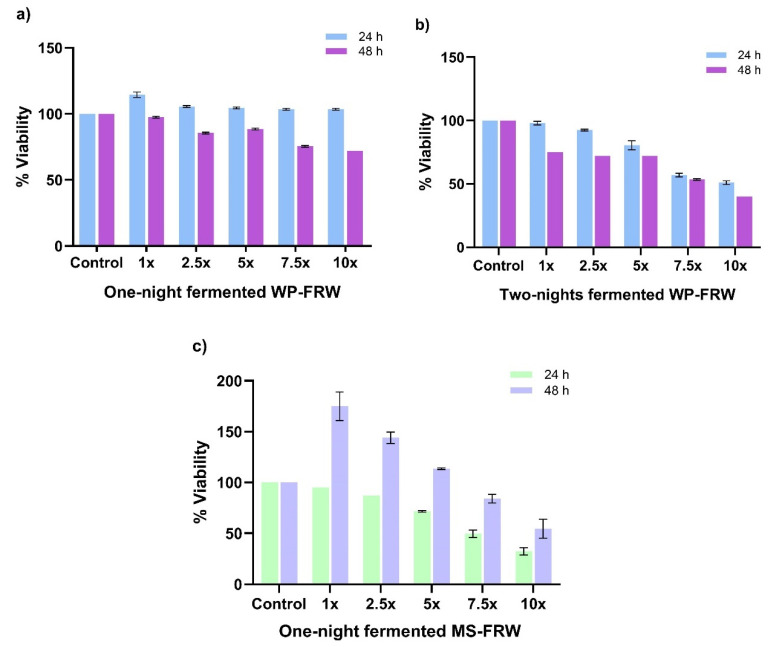
Effect of Fermented rice water (FRW) and Non-Fermented rice water (nFRW) treatment on the HT29 cell viability at 24 h and 48 h. (**a**) Overnight fermentation of WP-FRW. (**b**) Over two-nights fermentation of WP-FRW. (**c**) Overnight fermentation of MS-FRW. Results are an average of three independent experiments.

For the over-night fermented common rice variety WP-FRW from location 1, we observed an increase in the proliferation of the HT29 cells in all the concentrations tested for the 24 h treatment. However, there is an FRW concentration-dependent decrease in viability at 48 h (Figure 3a). For the two-nights-fermented WP-FRW, from another place (location 3), a concentration-dependent decrease in the viability was observed at both 24 h and 48 h treatments, without an increase in the proliferation at both time points (Figure 3a).

For the over-night fermented traditional red rice variety MS-FRW from location 4, there is a concentration dependent decrease in the cell viability at both 24 h and 48 h treatments. However, while the initial time point (24 h) of MS-FRW treatment does not show any increase in cell proliferation, there is a significant increase in the proliferation of colonocytes at lower concentrations, i.e., 1×, 2.5×, and 5× for the later time point (48 h).

#### 3.4.2. Probiotics Generated Biomolecules Aid in Improving Colonocyte Health

##### Postbiotics in FRW Upregulate Nutrient Absorptive Genes

**Figure 4:**
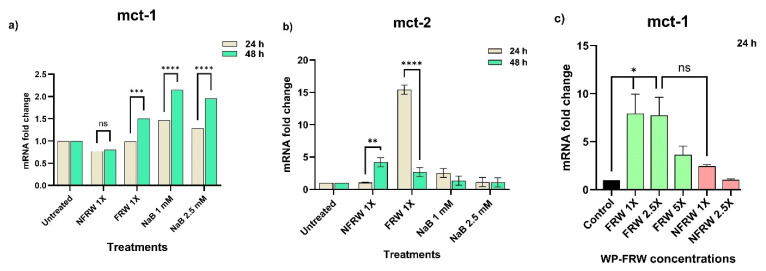
Role of Fermented rice water (FRW), Non-Fermented rice water (nFRW), and sodium butyrate (NaB) treatment (24 h and 48 h) on the gene expression of (**a**) monocarboxylate transporter -1 (mct-1) and (**b**) monocarboxylate transporter-2 (mct-2). Results are an average of two independent experiments, * *p* < 0.05; ** *p* < 0.01; *** *p* < 0.001; **** *p* < 0.0001. (**c**) Dose response of WP-FRW and WP-nFRW on the mct-1 gene expression at 24 h. Results are an average of three independent experiments, ** *p* < 0.01, ns = non-significant.

We assessed the ability of the fermented rice water of the commonly consumed rice variety (WP-FRW (neat)) on the expression of the monocarboxylate transporter mct-1 in HT29 cells (Figure 4a). We observed an increase in the mct-1 expression only at the 48 h time point. The positive control, where cells were treated with 1 mM and 2.5 mM sodium butyrate, showed a heightened expression of mct-1 as compared to the untreated control at both 24 h and 48 h treatments. We arrived at the above-mentioned concentrations for consideration as positive control after performing a sodium butyrate dose response proliferation assay for the HT29 cells (Figure S2). The matched non-fermented rice water treatment (WP-NFRW), which serves as an experimental control, did not elicit mct-1 expression at both time points (Figure 4a).

Under the same conditions, mct-2 expression was increased several folds (15-fold) at 24 h time point for the WP-FRW treatment (Figure 4b). Even though we observed an increased expression of mct-2 at 24 h, it was very much subdued at 48 h. Interestingly, we saw a 3-fold increase in mct-2 expression even for the WP-NFRW treatment at 48 h (Figure 4b).

To check if an increase in the concentration of the FRW might proportionately reflect the response in the expression of the transporter genes, we treated lyophilized WP-FRW reconstituted at various concentrations (1×, 2.5× and 5×). We observed a 3-to-7-fold increase in the mct-1 expression across all concentrations of WP-FRW, at the 24 h time point (Figure 4c). Interestingly, the WP-NFRW treatment caused a two-fold increase in mct-1 expression (Figure 4c).

##### Fermented Rice Water Modulates the Expression of Cytoskeletal Genes

Because expression of tight junction proteins by colonocytes are integral in maintaining intestinal barrier function, we assessed the ability of WP-FRW to modulate the expression of the tight junction genes occludin and zo-1 upon treatment for 24 h. The treatment of FRW at increasing concentrations showed heightened levels of gene expression for both genes: occludin (25- to 50-fold) (Figure 3a) and zo-1 (3- to 10-fold) (Figure 3b) as compared to the control non-fermented rice water (NFRW).

Since the HT29 cells display an undifferentiated (normal) and differentiated (absorptive) phenotype, we assessed the level of gene expression of the major cytoskeletal molecule, actin. The treatment of WP-FRW at increasing concentrations shows several hundred-fold increased levels of gene expression for actin (25- to 230-fold) as compared to the control non-fermented rice water (NFRW) (Figure 3c).

##### Fermented Rice Water Modulates the Metabolic Pathway

We observed a change in the WP-FRW treated samples for the expression of the central glycolytic pathway gene, gapdh. Although gapdh is a commonly used housekeeping gene, we observed several hundred-fold upregulation in gapdh expression in a concentration dependent manner with 1× concentration being the highest.

## 4. Discussion

In this study, we investigated naturally fermented rice varieties with respect to the environmentally dominated probiotics, macronutrient alterations, and their role in aiding colonocyte health using the colon cell line HT29.

Although fermented foods in general have been known to promote gut health, most studies have focused only on the probiotics present in the fermented foods as main players in bringing about this wellness state. Here, we wanted to investigate the functional role of rice-fermented postbiotics. Probiotic species use carbon in the carbohydrates of foods as their energy source; however, variations in the carbohydrates present in the foods allow for selection of unique probiotic species for fermentation, in nature. As a result, a unique synbiotic mixture is formed during the fermentation process. Consequently, the post-biotic composition also varies thus contributing to specific influences on the colonocytes.

Studies on rice and rice-based fermented beverages reveal biotransformation of the primary food source to beneficial secondary metabolites. While many food sources serve as the carbon source for fermentation, rice is a common and staple food that can be easily fermented without the need for industrial involvement. In this study, we show that simple natural fermentation allows for the enrichment of several probiotic species irrespective of the rice variety (Table 1). Fermentative facultative anaerobes were identified at four different locations of fermentation including domicile and Hospital environments. This shows that microcosms of fermented foods such as the FRWs can be generated overnight. Interestingly, we identified the LAB group of fermenters to predominate the fermented rice cultures (Table 1). The major LABs identified in our study, namely *Leuconostoc* and *Weisella* genus, are known to be present during the fermentation of rice and other forms of foods such as cereals and vegetables [43,44,45]. In general, Lactic Acid Bacteria (LAB) have been shown to modulate local and systemic effects in dysbiosis in mice [39,46,47]. Although there are many probiotic strains, a selective few, such as those of the genus *Lactobacillius* and *Bifidobacterium*, are renowned for their role in boosting gut health. While they may actively influence the gut health through their secondary metabolites, it is known that the domination of probiotics shifts the gut microbiota to a beneficial anti-opportunistic pathogenic environment [48]. Understandably, the synbiotic consortia of probiotics in the fermented rice water may also suppress the growth of commensals, enriching the health-beneficial biomolecules in FRW. Notably, fermentation of rice in domicile environments generated synbiotic mixtures that were dominated by the probiotic bacterial species (Table 1 and Table 2). We identified the probiotics present in FRWs by culturing them in the probiotic-selective microbial culture plates. However, we noticed that upon culturing the commercially available probiotic supplement (as a control), we were not able to isolate all the probiotic strains provided in the capsule (Table S1). This may be due to the inefficiency to revive the strains from the capsule or due to poor culture- selection conditions. Thus, it is possible that we may not have identified all the probiotic species present in our FRW samples as well. Moreover, there may be inherent rice-based natural selection of the fermenters. This is evident in the macronutrient analysis were we noticed that the high starch, low fibre white rice varieties (Raw Rice and White Ponni) generated more carbohydrates (NFE) in the FRWs as compared to their own non-fermented counterparts and the more fibrous, high-flavonoid-containing traditional rice variety, Mappillai Samba (Table 3).

The concentrations of the postbiotic-bioactives in each FRW sample may differ due to the differences in the fermenting bacterial species in addition to their bio load present during the fermentation process. Therefore, for functional analysis of FRW, we first studied the influence of the various varieties of fermented rice-derived postbiotics, i.e., the bioactives, on the proliferation of colonocytes at 24 h and 48 h time points post-treatment. We observed either marginal proliferation to no change in the growth of these cells at lower concentrations. However, the growth inhibition of HT29 cells at higher concentrations of the FRW (5×, 7.5× and 10×) may be due to interference-induced inhibitory effect of the biomolecules and/or concentration-dependent toxicity (Figure 3).

**Figure 5:**
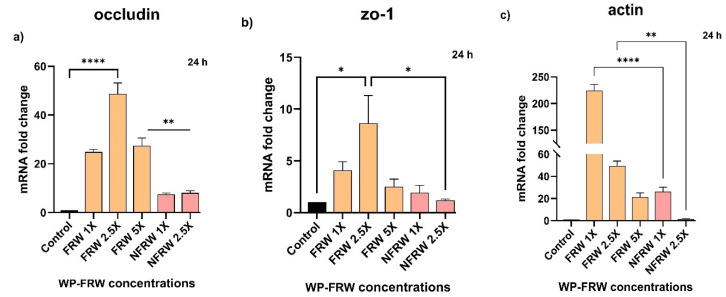
Gene expression analysis of the cytoskeletal genes (**a**) occludin, (**b**) ZO-1, and (**c**) β-actin at 24 h of WP-FRW and WP-NFRW treatment in the HT29 cells. Results are an average of three independent experiments, * *p* < 0.05; ** *p* < 0.01; **** *p* < 0.0001.

Colonocyte health can be assessed on a cellular metric that includes maintenance of a general epithelial morphology, ability to form an effective lining of the intestine, possess good absorptive, transporting, and secretory capabilities, elicit a defensive response to toxic stimulus and maintenance of the cellular integrity. Therefore, we tested the role of FRW on biological indicators of a few of these metrics. Since probiotics produce various secondary metabolites such as short-chain-fatty acids during the fermentation process, we checked if the colonocytes transform to an absorptive phenotype in response to the micronutrients-enriched FRW. Because short-chain -atty acids are the main source of energy for colonocytes, we checked for changes in the expression of the monocarboxylate transporters mct-1 and mct-2 that are involved in the transport of metabolites such as lactate, acetate, pyruvate, butyrate, and ketone bodies. Our results show that FRW significantly induces the expression of mct-1 at lower and higher concentrations, indicating a need to increase this classic short-chain-fatty acid transporter (Figure 4a,c). The positive control samples (1 mM and 2.5 mM sodium butyrate treated) show an upregulation of mct-1, indicating the positive responsiveness of HT29 cells to the SCFA, butyrate. The upregulation of another isoform mct-2 in the FRW sample (Figure 4b) shows that this transporter may preferentially transport non-butyrate SCFAs present in the FRW. Also, it is possible that the postbiotic biomolecules present in FRW may act synergistically to upregulate more than one SCFA transporter (Figure 4). The upregulation of mct-1 upon WP-NFRW treatment at 24 h may be due to the micronutrients leached off from the cooked rice. Nevertheless, the effect of WP-FRW is significant compared to WP-NFRW (Figure 4c). Likewise, we observed significant upregulation of the genes encoding for the tight junction proteins occluding and zo-1 in the FRW treatment as compared to the non-fermented (NFRW) and the untreated control (Figure 5a,b). This is indicative that the postbiotics in FRW may contain bioactives that, either directly or indirectly, allow transformation of the undifferentiated HT29 phenotype to an absorptive columnar phenotype, since these proteins are present at the apical side of the cell. Interestingly, there is a corroborating increase (several hundred-fold) in the actin gene expression in the FRW treated samples (Figure 5c), also supportive of the role of FRW in contributing toward strengthening structural integrity of the colonocytes.

**Figure 6:**
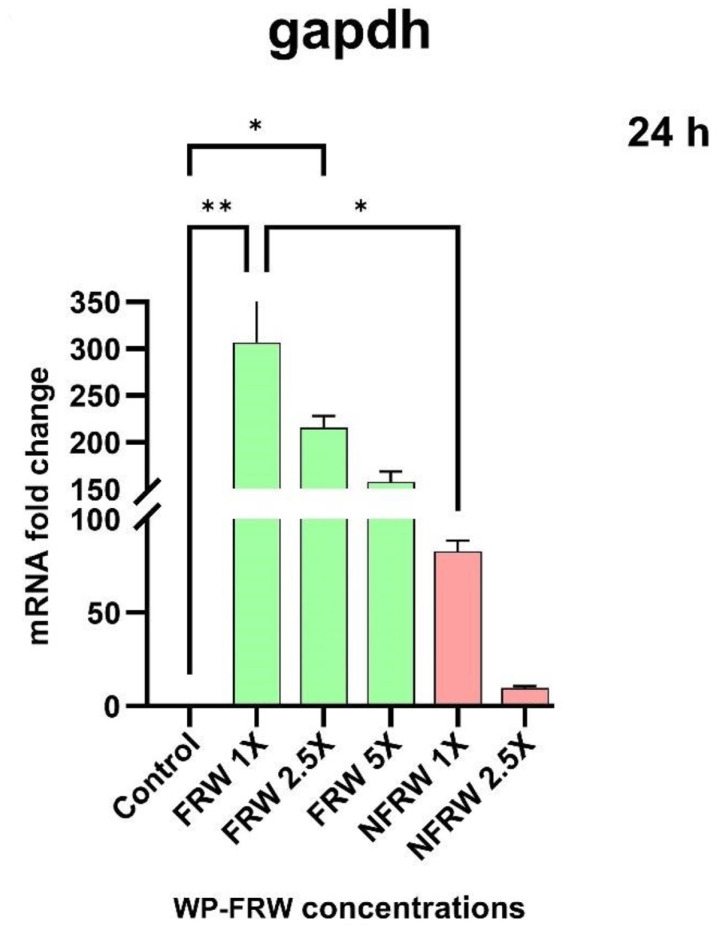
Dose response effect of WP-FRW and WP-NFRW on the GAPDH gene expression in HT29 cells at 24 h. Results are an average of three independent experiments, * *p* < 0.05; ** *p* < 0.01.

Furthermore, we noticed an increase (several hundred-fold) in the gapdh gene (normally considered as reference/housekeeping gene) expression in the FRW treatment, as compared to the NFRW and untreated control sample (Figure 6). This indicates a need for upregulating the glycolytically active metabolic pathway. This could be due to the increased concentrations of the monosaccharides present in FRW as a result of the fermentation process. There may also be a demand for increased cellular energy requirement to carry out the energy expensive structural alterations involved during differentiation of the cells towards an absorptive phenotype. This combined with the fact that the HT29 cells consume higher amounts of glucose may explain the upregulated gapdh gene expression. It remains to be understood if a parallel β-oxidation metabolic pathway, the major energy producing pathway for colonocytes, is also upregulated.

Our study identifies a rich and diverse range of postbiotic metabolites even in the short duration of simple natural fermentation of edible rice (Figure 2A and Table S2). Recent studies on fermented foods by the LAB group have also reported the presence of polyphenols (4-hydroxyphenyl propionic acid) and indoles that act as anti-oxidants and anti-inflammatory agents, which improve immunity and gut barrier homeostasis [40,49,50,51,52]. Indeed, the most predominant metabolite in our study is lactic acid: a result of the fermentation of rice by the LAB group bacteria. Lactic acid is also known to be anti-hypertensive, in addition to being bifidogenic [38,53,54]. Fermentation of polysaccharides has shown to produce Betaine, L-carnitine, and amino imidazole all of which upregulate the tyrosine and tryptophan biosynthesis pathways [55]. These pathways mitigate inflammation (Figure S6) and increase intestinal integrity. Thus, our study identifies previously reported biomolecules of fermentation [55] (Table S2). In addition, we report a consortium of postbiotics that may synergistically act on the gut to render its benefits.

The authors do identify some limitations in the study. Since the synbiotics generated during natural fermentation of various types of rice as prebiotics greatly depend on the environment, it is not possible to control for the quantities and/or identities of the bioactives obtained. Therefore, functional effects can only be generalized to FRW as a whole rather than to one or more of their specific metabolites. Given that the functional assays were performed with the FRW-bioactives, it would complement the study to identify the metabolites in the FRW. Although the cancer cell line HT29 serves as a model system for nutrient absorption studies, delineation of the molecular mechanisms specific to the probiotic biomolecules of FRW might need investigations of the same in a normal intestinal cell line. The gene expression results are indicators of the change in cellular physiology, but corroboration of the same at the functional level may underscore the observations.

To summarize, we show that despite the differences in the environmental conditions, the variations in rice varieties and their fermenting probiotic bacteria, the postbiotics obtained via rice fermentation nevertheless are beneficial for colonocyte health.

## 5. Conclusions

This study is the first of its kind to demonstrate as a proof of principle that postbiotics of naturally fermented rice water positively modulates colonocyte health. The study also shows that fermentation of one of the food staples viz rice, enriches probiotic bacteria and their biomolecules. Edible rice serves as a simple prebiotic for the fermenting probiotic organisms, generating up to billions of CFUs per ml in just a short duration (overnight). Fermentation-derived postbiotic biomolecules elicit nutrient absorptive, barrier strengthening, and energy-expensive molecular signatures indicative of improved colonocyte health in vitro. Overall, our results suggest that FRW acts as a multifunctional synbiotic, promote growth of a mixture of probiotics and enrich bioactives which upon chronic consumption, can restore gut homeostasis.

## Figures and Tables

**Table 1:** Probiotic species identified in various samples of fermented rice water.

S. No	Rice Variety	Location	Probiotic Bacteria Identified	CFU/mL
1.	Raw Rice	Location 2	*Lactococcus lactis* ssp. *lactis*	54 × 10^9^
			*Weisella confusa*	
		Location 4	*Leuconostoc lactis*	47.4 × 10^6^
2.	White Ponni	Location 3 (over two-nights fermented)	*Lactococcus lactis*	85.5 × 10^9^
			*Lactobacillus nagelii*	
			*Lactobacillus delbrueckii* ssp. *indicus*	
		Location 3 (overnight fermented)	*Leuconostoc lactis*	UD
			*Lactobacillus fermentum*	
		Location 1	*Leuconostoc lactis*	15 × 10^8^
			*Weisella confusa*	50 × 10^6^
		Location 4	*lactococcus taiwanensis*	1.28 × 10^5^
			*Lactococcus lactis*	
3.	Mappillai Samba ^#^	Location 4	*Leuconostoc lactis*	6.3 × 10^8^
			*Weisella cibacria*	
			*Weisella confusa*	
4.	Control *	Location 4	*Lactobacillus plantarum*	UD

* Control: Commercially available probiotic supplement tablet (Fourrts^TM^). ^#^: Traditional rice variety. UD: undetermined.

**Table 2:** Commensals and opportunistic pathogens identified.

S. No	Rice Variety	Location	Other Bacteria
1.	Raw rice	Location 4	*Streptococcus equinus*
			*Acinetobacter baumannii*
			*Franconibacter pulveris*
2	White Ponni	Location 1	*Acinetobacter baumannii*
			*Streptococcus lutetiensis*
			*Sterptococcus infantaurius*
			*Kocuria kristinae*
		Location 4	*Franconibacter pulveris*
			*Enterobacter cloacae*
			*Acinetobacter baumannii*
			*Cronobacter* sp.
3	Mappillai Samba	Location 4	*Klebsiella penumoniae* ssp. *pneumoniae*
			*Franconibacter pulveris*
			*Bacillus cereus*
			*Enterococcus gallinarum*
			*Sterptococcus infantaurius* ssp. *infantarius*
			*Enterobacter cloacae complex*
4	Control	Location 4	*Enterococcus faecium*

NB: Location 4—Hospital environment; Location 1—Domicile environment.

**Table 3:** Principle component analysis of pre-and post-fermented rice variety and its water.

S. No	Rice Varieties	Rice/ Rice Water	Types of Fermentation	Moisture (%)	Dry Matter Basis (%)				
					Crude Protein	Crude Fibre	Ether Extract	Total Ash	Nitrogen Free Extract (NFE)
1.	Raw rice *	Rice	Before fermentation	80.9	8.55	0.16	0.22	0.28	90.79
			Non-fermentation	80.63	8.10	0.18	0.31	0.26	91.15
			After fermentation	79.9	8.6	0.19	0.38	0.26	90.57
		Rice water	Before fermentation	95.69	6.51	Not traceable	0.29	0.46	92.74
			Non-fermentation	96.44	6.88	Not traceable	0.14	0.72	92.26
			After fermentation	96.0	5.98	Not traceable	0.14	0.62	93.26
2.	Mappillai samba	Rice	Before fermentation	80.45	9.18	0.48	0.98	0.58	88.78
			Non-fermentation	85.18	8.5	0.52	1.04	0.55	89.39
			After fermentation	85.42	8.87	0.6	1.25	0.47	88.81
		Rice Water	Before fermentation	98.19	4.21	Not traceable	0.34	1.45	94.0
			Non-fermentation	98.27	4.45	Not traceable	0.20	1.59	93.76
			After fermentation	97.4	4.77	Not traceable	0.19	1.61	93.43
3.	Parboiled rice *	Rice	Before fermentation	81.96	8.62	0.31	0.50	0.46	90.11
			Non-fermentation	80.19	8.59	0.46	0.46	0.54	89.95
			After fermentation	81.5	9.05	0.28	0.58	0.50	89.59
		Rice Water	Before fermentation	97.02	6.01	0.21	0.36	1.81	91.61
			Non-fermentation	99.03	7.30	0.25	0.54	2.72	89.19
			After fermentation	96.73	4.97	Not traceable	0.28	1.78	92.97

* Identities of rice varieties are unknown as they were sourced from a public rice repository.

## Data Availability

Raw data files for metabolomic analysis of the fermented rice water can be obtained upon request to the corresponding author.

## Supplementary Materials

The following supporting information can be downloaded at: https://www.mdpi.com/article/10.3390/biom14030344/s1, Figure S1: Dose response study of sodium butyrate (NaB) on the cell viability at 24 and 48 h in HT 29 cells using MTT. Figure S2: Different varieties of rice used in this study for the fermentation process. Figure S3: Post-fermentation of rice in the absence and the presence of fermenters illustrates the post-fermentation of rice wherein air pockets are seen in fermenter confirming the fermentation process. No such air pockets observed in non-fermenter, indicating the inhibition of the fermentation process by the use of antibiotics (pen/strep) and sodium azide. Figure S4: Heatmap showing the hierarchical clustering of top 100 metabolites from the total metabolites present in one overnight fermented and over two nights fermented rice water (FRW) of white Ponni (WP) rice variety. The colour scale (blue to red) represents the relative abundance of each metabolite. Figure S5. Histogram representations of the top three significantly up-, down- regulated metabolites in the overnight fermented (White Ponni WP) over the over two-nights fermented rice water. Table S1: Pre- and Probiotic composition of PrePro capsule ^®^ Fourrts. Figure S6. Effect of FRW and NFRW on the inflammatory molecule PTGS2 in LPS stimulated HT29 cells. Results show a single experiment of the quantitative ELISA for the levels of PTGS2 upon treatment of the White Ponni (WP) (common rice) and Mappillai Samba (MS) (traditional rice) varieties. Table S2. WP-Fermented Rice Water metabolites (top 100) categorized based on their biological function.
