# Supplementary material for: Postbiotics of Naturally Fermented Synbiotic Mixture of Rice Water Aids in Promoting Colonocyte Health
Source: Biomolecules. 2024 Mar 13;14(3):344. doi: 10.3390/biom14030344 (PMC10968502; doi:10.3390/biom14030344)
Supplement: Supplementary file 1 [file biomolecules-14-00344-s001.zip › biomolecules-2777865-supplementary.pdf]

Supplementary material

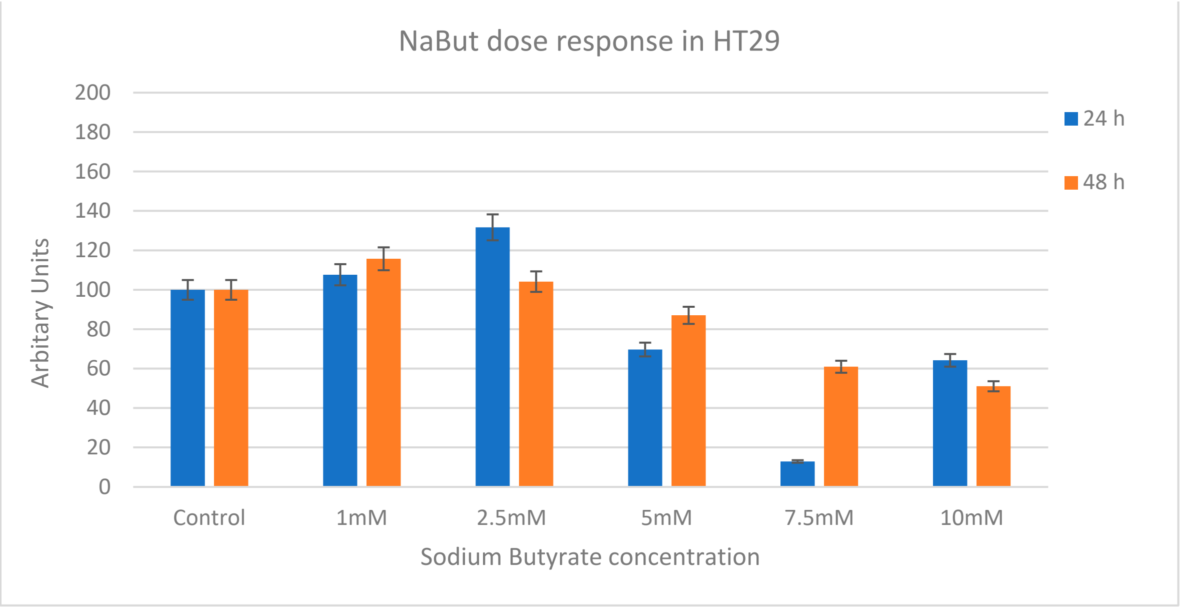

Fig. S1. Dose response study of sodium butyrate (NaB) on the cell viability at 24h and 48h in HT 29 cells using MTT

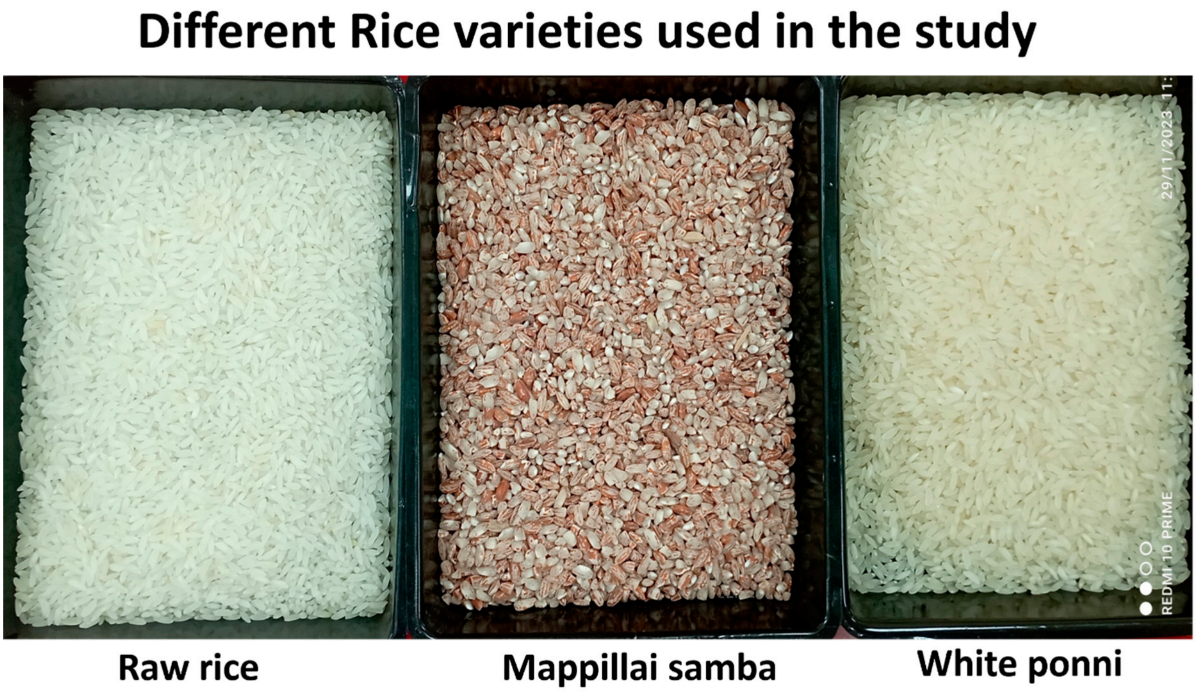

Fig. S2. Different varieties of rice used in this study for the fermentation process

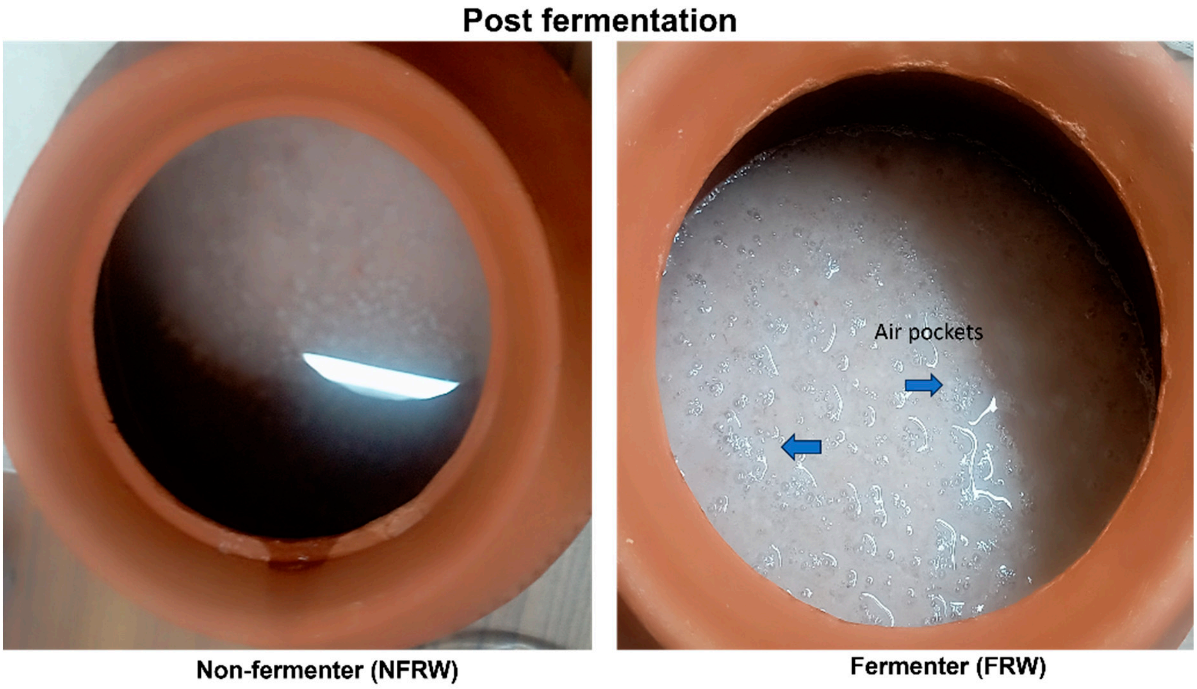

Fig. S3. Post fermentation of rice in the absence and the presence of fermenters illustrates the post-Fermentation of rice wherein air pockets are seen in fermenter confirming the fermentation process. No such air pockets observed in non-fermenter indicating the inhibition of the fermentation process by the use of antibiotics (penicillin/streptomycin) and sodium azide.

Table S1: Pre- and Probiotic composition of PrePro capsule ® Fourrts

**S.NO**	**Isolate/ Nutrition Name**	**Volume**	**Unit**
1.	Lacto bacillius acidophilus	200	Million cfu
2.	Lacto bacillius rhamnosus	100	Million cfu
3.	Lacto bacillius casei	100	Million cfu
4.	Lacto bacillius bulgaricus	100	Million cfu
5.	Lacto bacillius plantarum	100	Million cfu
6.	Bifidobacterium longum	100	Million cfu
7.	Bifidobacterium breve	100	Million cfu
8.	Bifidobacterium infantis	100	Million cfu
9.	Streptococcus thermophilus	100	Million cfu
10.	Fructo-oligosaccharide	100	mg
11.	Betaglucan	50	mg

Heat map of the top 100 Postbiotic metabolites of the one-night vs two-nights fermented rice water

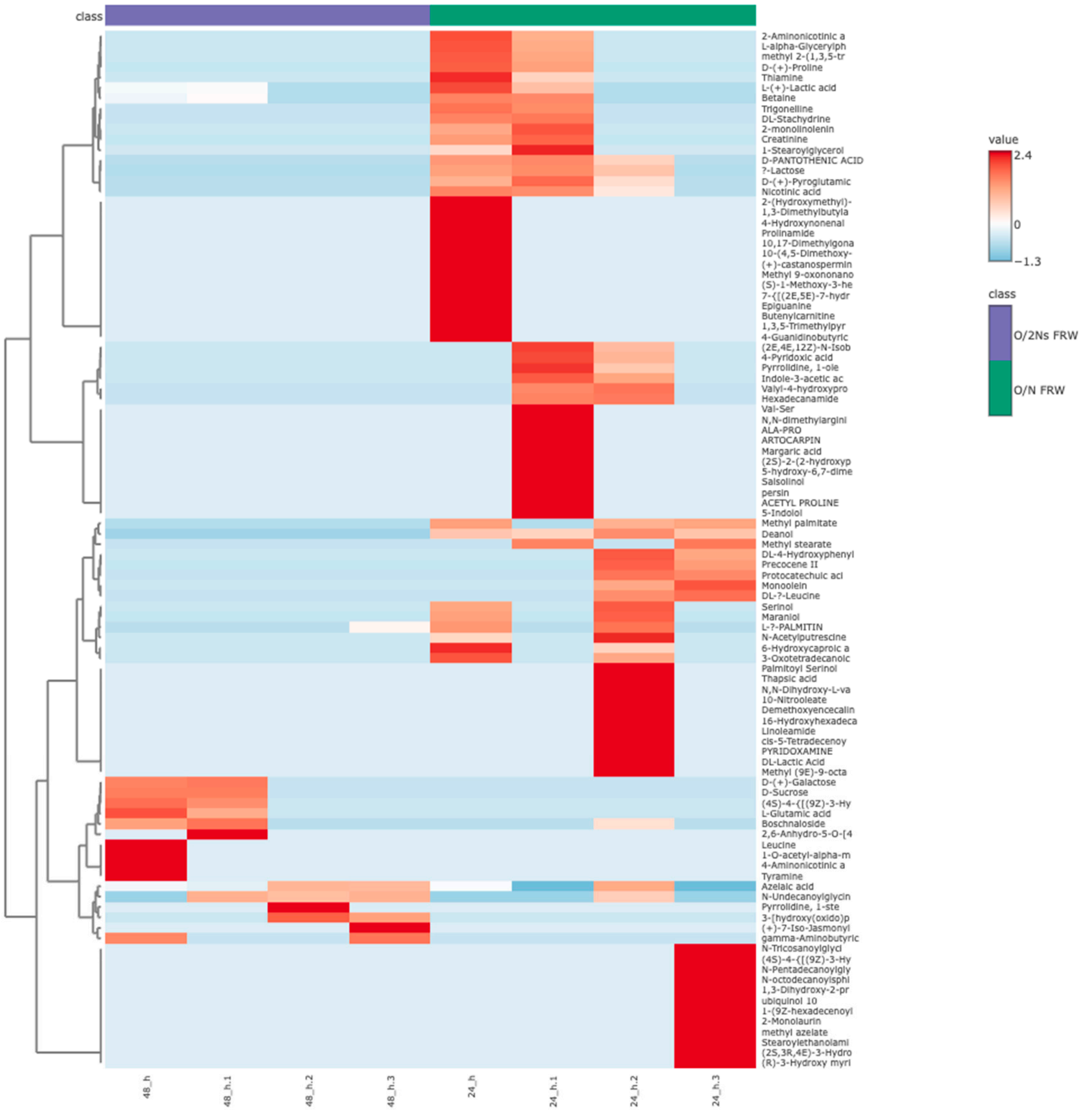

Fig. S4. Heatmap showing the hierarchical clustering of top 100 metabolites from the total metabolites present in one overnight fermented and over two-nights fermented rice water (FRW) of white Ponni (WP) rice variety. The colour scale (blue to red) represents the relative abundance of each metabolite.

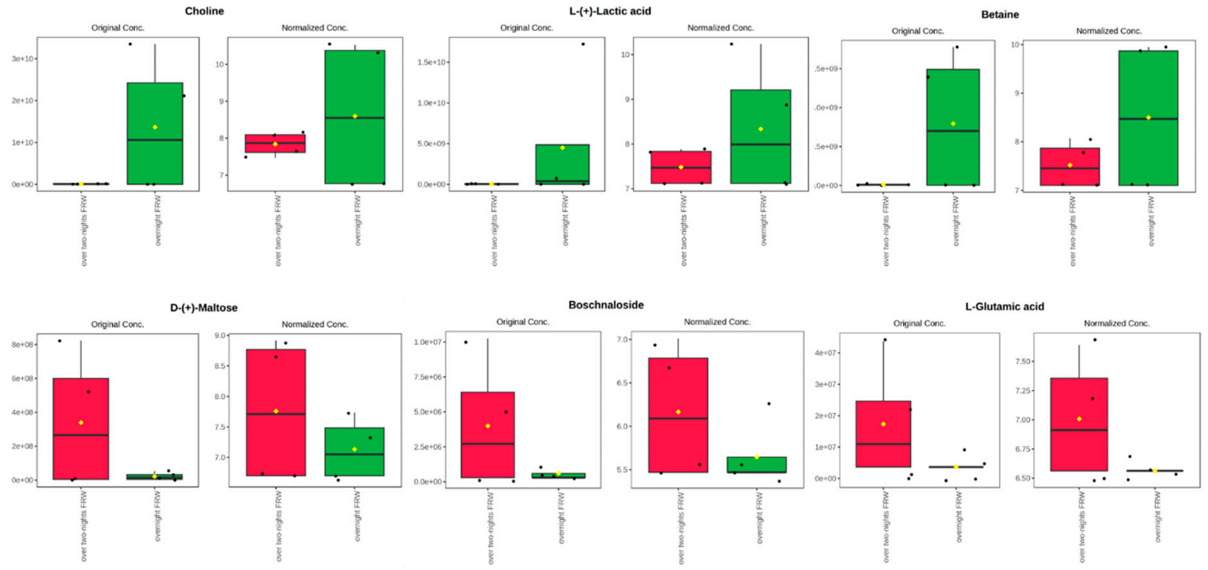

Fig. S5. Histogram representations of the top three significantly up-, down- regulated metabolites in the overnight fermented (White Ponni WP) over the over two-nights fermented rice water.

Table S2. WP-Fermented Rice Water metabolites categorized based on their biological function.

S.No	Energy/ Metabolism	Microbial postbiotics	Phytoactive - postbiotics	Postbiotic byproducts	Anti-oxidants/-inflammatory/-cancer/-hypertensive/-microbial/ neuroprotective
1.	Palmitic Acid	Choline	castanospermine	Levulinic acid	Acetylagmatine
2	Butenylcarnitine	L-alpha-Glycerylphosphorylcholine	Sinapine	Pivagabine	N-Acetylputrescine
3	4-Guanidinobutyric acid	1-Stearoylglycerol	7-{[(2E,5E)-7-hydroxy-3,7-dimethylocta-2,5-dien-1-yl]oxy}-2H-chromen-2-one (auraptene)	2-(Hydroxymethyl)-1-methyl-3,4,5-piperidinetriol	2-Aminonicotinic acid
4	11-Aminoundecanoic acid	Choline O-Sulfate	persin	3-Pentyl-5-isoxazolamine	Azelaic acid
5	Methyl palmitate	Methyl stearate	Indoline	Methyl 9-oxononanoate	8-AzaG
6	(1S,2R,5S)-2-Isopropyl-5-methylcyclohexyl 3-oxobutanoate	A-12(13)-EpODE	Morusinol	1-(4-Methyl-1-piperazinyl)ethanone	Epiguanine

7	Methyl palmitate	sphinganine	Methyl nigakinone (mushrooms)	(S)-1-Methoxy-3-heptanethiol	Trigonelline-legumes seeds
8	Thiamine	12-Hydroxylauric acid	Coumarin (flavour industries)	2-Hydroxypropyl methacrylate	DL-Stachydrine
9	4-Hydroxynonenal	16-Hydroxyhexadecanoate	Artocarpin	2,3-dihydro-2-spiro[1-(benzyl) piperidin-4-yl]-1,3-benzothiazole	Serinol
10	NP-021797 ((Z)-3-hydroxydodec-5-enoic acid)	Threo-Sphingosine	Embelin	Diethylene glycol n-butyl ether	Maraniol
11	DL-Carnitine	Stearic acid	Precocene II	10,17-Dimethylgona-4,13(17)-dien-3-one	Pyrrolidine, 1-oleoyl-
12	Desthiobiotin	N-octodecanoylsphinganine	homodihydrocapsaicin-I	2-Pyridinol, 1-acetyl-3,6-bis(tert-butylthio)-1,2,3,6-tetrahydro-, acetate (ester)	3-Methyladenine
13	Prolylleucine	(4S)-4-{[(9Z)-3-Hydroxy-9-hexadecenoyl] oxy}-4-(trimethylammonio)butanoate	Demethoxyencecalin	methyl 2-(1,3,5-trihydroxy-4a-methyl-8-oxo-decahydronaphthalen-2-yl) prop-2-enoate	N,N-dimethylarginine
14	2,3,4,9-Tetrahydro-1H-beta-carboline-1,3-dicarboxylic acid	1-(9Z-hexadecenoyl)-2-(9Z,12Z-octadecadienoyl)-sn-glycerol	Lauramide	1H-indene-3-carboxamide	Acetyl Proline
15	D-Pantothenic Acid	N-eicosanoylsphinganine	Thapsic acid	2-(Carboxyacetamido)benzoic acid	Val-Ser
16	D-(+)-Pyroglutamic Acid	Prolinamide	Boschnaloside	1,3-Dimethylbutylamine	1-Hexadecanoylpyrrolidine
17	Nicotinic acid	10-(4,5-Dimethoxy-2-methyl-3,6-dioxo-1,4-	sinapoylglucose	tranexamic acid	Indoline

		cyclohexadien-1-yl) decanal			
18	D- (+)-Proline	Benzoic acid	Ostruthin	5-Indolol	ALA-PRO
19	Lactose		3-(3,4-dihydroxyphenyl) propanoic acid	Deazaflavin	(2S,3S)-2,3-dihydro-3-hydroxyanthranilic acid zwitterion
20	Betaine		2-Monolaurin	Erucamide	(2E,4E,12Z)-N-Isobutyl-2,4,12-octadecatrienamide
21	D-Glucose 6-phosphate		L-(+)-Erythrulose	3-amino-1H-pyrazolo[4,3-c] pyridine-4,6-diol	2-Hydroxyethyl 12-hydroxyoctadecanoate
22	trimethyl-1-cyclohexen-1-yl)-3-buten-2-one (EN0350000)		(+/-)-nicotine	(2S)-2-(2-hydroxypropan-2-yl)-2H,3H,7H-furo[3,2-g] chromen-7-one	Pyrogallol
23	3-Oxotetradecanoic acid		(+)-7-Iso-Jasmonyl-L-isoleucine	5-hydroxy-6,7-dimethoxy-2-phenyl-4H-chromen-4-one	L-Pyroglutamic acid
24	L-Palmitin			Arachidoyl Ethanolamide	7-Aminomethyl-7-deazaguanine
25	1-Stearoylglycerol			Methyl (6E)-2,6-dimethyl-10-(4-methyl-1-piperazinyl)-10-oxo-6-decenoate	Valyl-4-hydroxyproline
26	2-monolinolenin			Palmitelaidic acid methyl ester	Allantoin
27	6-Hydroxycaproic acid			N-(9-oxodecyl) acetamide	A-12(13)-EpODE
28	L-(+)-Lactic acid			Glycidyl Stearate	D-ribosylnicotinate
29	Methylmalonic acid			2-[(N-Benzoylphenylalanyl)amino]-3-	Proline

				phenylpropyl acetate	
30	D-(+)-Maltose			1,3-Dihydroxy-2-propanyl icosanoate	Hexadecanamide
31	Choline O-Sulfate			Bis(4-ethylbenzylidene) sorbitol	Panthenol
32	Methyl stearate			[(2R,3S,4S,5R,6R)-6-{4-[(4,5-dihydroxy-6-{[(4-hydroxy-2-methylidenebutanoyl)oxy]methyl}oxan-3-yl)oxy]-3-methylidene-4-oxobutoxy}-3,4,5-trihydroxyoxan-2-yl]methyl 4-hydroxy-2-methylidenebutanoate	sphinganine
33	Pyrrolidine, 1-oleoyl-			2,6-Anhydro-5-O-[4-({6-O-[4-(D-glucopyranosyloxy)-2-methylenebutanoyl] -D-glucopyranosyl} oxy)-2-methylenebutanoyl] -1-O-(4-hydroxy-2-methylenebutanoyl) hexitol	13S-hydroxyoctadecadienoic acid
34	2-(alpha-D-mannosyl)-D-glyceric acid			Creatinine	Threo-Sphingosine

35	1-Hexadecanoylpyrrolidine			Glycoursodeoxycholic acid 3-sulfate	N-Undecanoylglycine
36	(2S,3S)-2,3-dihydro-3-hydroxyanthranilic acid zwitterion			5-Methoxy-3-indoleaceate	Palmitoyl Serinol
37	(2E,4E,12Z)-N-Isobutyl-2,4,12-octadecatrienamide			Linoleamide	2,3,22,23-Tetrahydroxyergostan-6-one
38	Acetylcarnitine			Suberic acid	N,N-Dihydroxy-L-valine
39	D-ribosylnicotinate			N-Pentadecanoylglycine	Protocatechuic acid
40	Margaric acid			2-(14,15-Epoxyeicosatrienoyl) glycerol	20-Hydroxy (5Z,8Z,11Z,14Z) -eicosatetraenoic acid
41	D-Raffinose			Monoolein	12-Oxo phytodienoic acid
42	2-(methylthio)nicotinamide				DL-4-Hydroxyphenyllactic acid
43	N-({(1R,2S)-2-[(2Z)-5-Hydroxy-2-penten-1-yl]-3-oxocyclopentyl} acetyl) -L-isoleucine				L-Tyrosine methyl ester
44	L-(+)-Leucine				2-Arachidonyl glycerol ether
45	12-Hydroxylauric acid				Leucine
46	16-Hydroxyhexadecanoate				10-Nitrooleate
47	g-Butyrobetaine				2-Isopropylmalic acid
48	cis-5-Tetradecenoylcarnitine				Nicotinuric acid
49	N-(2,6,10,14-Tetramethylpentadecanoyl) glycine				Militarinone A
50	O-heptanoylcarnitine				N-octodecanoylsphinganine
51	Stearic acid				Pentadecanoyl Ethanolamide

52	Methyl (9E)-9-octadecenoate				Spergualin
53	4-(2-Carboxyethyl)-2-methoxyphenyl beta-D-glucopyranosiduronic acid				(4S)-4-{[(9Z)-3-Hydroxy-9-hexadecenoyl]oxy}-4-(trimethylammonio)butanoate
54	Pyridoxamine				1-(9Z-hexadecenoyl)-2-(9Z,12Z-octadecadienoyl)-sn-glycerol
55	DL-Lactic Acid				N-eicosanoylsphinganine
56	3-Phenyllactic acid				methyl azelate
57	Oleic acid alkyne				Stearoyl Ethanolamide
58	DL-Leucine				Tyramine
59	Nicotinuric acid				1,2-dithiole-3-thione
60	(R)-3-hydroxybutyrylcarnitine				Acetyl-methylcholine
61	Pentadecanoyl Ethanolamide				L-alpha-Glycerylphosphorylcholine
62	ubiquinol 10				Deanol
63	(4S)-4-{[(9Z)-3-Hydroxy-9-hexadecenoyl] oxy}-4-(trimethylammonio)butanoate				Choline
64	1-(9Z-hexadecenoyl)-2-(9Z,12Z-octadecadienoyl)-sn-glycerol				Pyrrolidine, 1-oleoyl-
65	2-Amino-3-hydroxyoctadecyl beta-D-glucopyranoside				DL-Phenylalanine
66	N-Tricosanoylglycine				(2E,4E,12Z)-N-Isobutyl-2,4,12-octadecatrienamide
67	3-(Icosanoyloxy)-4-(trimethylammonio)butanoate				Acetylcarnitine

68	(2S,3R,4E)-3-Hydroxy-2-(palmitoylamino)-4-octadecen-1-yl 5-acetamido-3,5-dideoxy-6-[(1R,2R)-1,2,3-trihydroxypropyl]-beta-L-threo-hex-2-ulopyranonosyl-(2->3)-beta-D-galactopyranosyl-(1->4)-beta-D-gluco				L-Pyroglutamic acid
69	(R)-3-Hydroxy myristic acid				N-Acetyl-L-leucine
70	3,4-Dihydroxyphenyl propionic acid				Proline
71	L-Glutamic acid				4-Pyridoxic acid
72	D-(+)-Mannose				Hexadecanamide
73	D-(+)-Galactose				Salsolinol
74	Leucine				Heptadecanoyl Ethanolamide
75	4-Aminonicotinic acid				Pyridoxamine
76	(4S)-4-{[(9Z)-3-Hydroxy-9-octadecenoyl] oxy}-4-(trimethylammoni o)butanoate				DL-4-Hydroxyphenyllactic acid
77	D-Sucrose				2-Arachidonyl glycerol ether
78	1-O-acetyl-alpha-maltose				Pentadecanoyl Ethanolamide
79	3-[2-(1,3-Benzodioxol-5-yl)-7-methoxy-1-benzofuran-5-yl]-3-hydroxypropyl hexopyranoside				Stearoylethanolamide
80	Guanine				gamma-Aminobutyric acid
81	8-Oxo-9-(3,4,5-trimethoxyphenyl)-5,5a,6,8,8a,9-hexahydrofuro [3',4':6,7] naphtho [2,3-d] [1,3] dioxol-5-yl hexopyranoside				γ-Aminobutyric acid (GABA)

82	3-[hydroxy(oxido)phosphoranyl] pyruvic acid				DL-tyrosine
83	Pyrrolidine, 1-stearoyl-				
84	Pyruvic acid				
85	2,3-Dihydroxypropyl stearate				

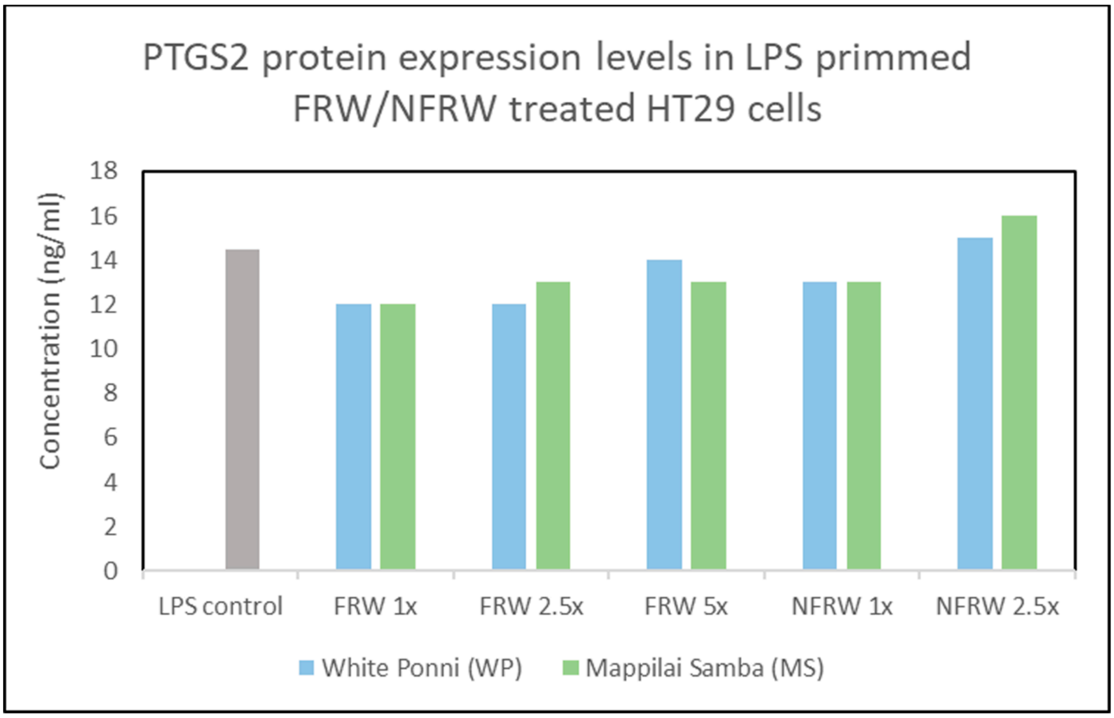

Fig. S6. Effect of FRW and NFRW on the inflammatory molecule PTGS2 in LPS stimulated HT29 cells. Results show a single experiment of the quantitative ELISA for the levels of PTGS2 upon treatment of the White Ponni (WP) (common rice) and Mappillai Samba (MS) (traditional rice) varieties.
